# Development of the Gemini Gel-Forming Surfactant with Ultra-High Temperature Resistance to 200 °C

**DOI:** 10.3390/gels8100600

**Published:** 2022-09-20

**Authors:** Peng Liu, Caili Dai, Mingwei Gao, Xiangyu Wang, Shichun Liu, Xiao Jin, Teng Li, Mingwei Zhao

**Affiliations:** 1 Shandong Key Laboratory of Oilfield Chemistry, Department of Petroleum Engineering, China University of Petroleum (East China), Qingdao 266580, China; 2 Key Laboratory of Unconventional Oil & Gas Development, China University of Petroleum (East China), Ministry of Education, Qingdao 266580, China

**Keywords:** clean fracturing fluid, VES, surfactant gel, rheology behavior, thermal stability

## Abstract

In order to broaden the application of clean fracturing fluid in ultra-high temperature reservoirs, a surfactant gel for high-temperature-resistant clean fracturing fluid was developed with a gemini cationic surfactant as the main agent in this work. As the fracturing fluid, the rheological property, temperature resistance, gel-breaking property, filtration property, shear recovery performance and core damage property of surfactant gel were systematically studied and evaluated. Results showed the viscosity of the system remained at 25.2 mPa·s for 60 min under a shear rate of 170 s^−1^ at 200 °C. The observed core permeability damage rate was only 6.23%, indicating low formation damage after fracturing. Due to micelle self-assembly properties in surfactant gel, the fluid has remarkable shear self-repairability. The filtration and core damage experimental results meet the national industry standard for fracturing fluids. The gel system had simple formulation and excellent properties, which was expected to enrich the application of clean fracturing fluid in ultra-high temperature reservoirs.

## 1. Introduction

**Figure 1:**
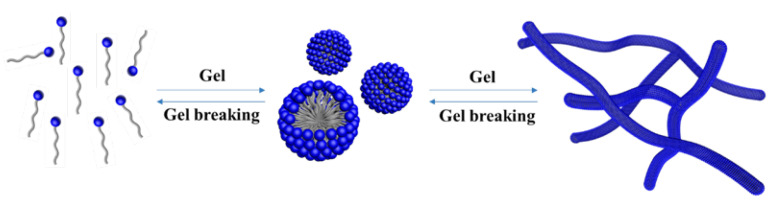
Mechanism of VES as fracturing fluid thickener.

In recent years, low permeability reservoir has become an important strategic energy resource [1]. However, these reservoirs have the characteristics of low permeability, which makes the development of these reservoirs challenging. Asphaltene control technology can reduce the damage of deposited asphaltenes to reservoir permeability and increase oil production [2,3]. Hydraulic fracturing technology is an important method to improve the production capacity of low-permeability oil reservoirs [4,5]. Fracturing fluid is the key factor of fracturing success. In the past few decades, soluble polymers, such as guar gum and acrylamide polymer, have been widely used as thickeners in fracturing fluids [6,7,8]. However, with the gradual transfer of oilfield development to deeper and tight reservoirs, decreasing the reservoir damage rate becomes particularly important [9,10]. Traditional polymer fracturing fluid easily produces insoluble residues after gel breaking, which will block the oil and gas migration channel and lead to low production of oil wells. Thus, low-damage fracturing fluid is paid more and more attention in low permeability and tight reservoir development [11,12]. As shown in Figure 1, viscoelastic surfactant (VES) can spontaneously form viscoelastic surfactant gel in aqueous solution to increase the viscosity of the solution, which are generally called “living polymer systems” [4,13,14,15,16]. In recent years, viscoelastic surfactant gels have often been used as clean fracturing fluids [17]. The viscoelastic surfactant gel without residue after gel breaking only generates a little reservoir damage [18,19]. In addition, the gel breaking fluid has good imbibition and oil expulsion characteristics, which has attracted the attention of the oil industry and research institutes in recent years [20,21,22].

At present, VES for viscoelastic surfactant gel mainly includes anionic VES, cationic VES and zwitterionic VES, among which the most widely used is cationic VES [23,24,25]. The poor temperature resistance of commonly used single-chain surfactants limits their application in deeper and higher temperature reservoirs [26,27]. Therefore, it is urgent to develop high-temperature-resistant viscoelastic surfactant gels. Improving the temperature resistance can be achieved by increasing the chain length of surfactant, introducing some special molecular structures and surfactant mixture [28,29,30]. The hydrogen bond interaction produced by amide structure can enhance the structure of micelles to a certain extent, thereby improving the temperature resistance of VES [28]. The introduction of hydroxyl into the molecular structure of surfactant can enhance the thermal stability of micelle structure and improve the temperature resistance of clean fracturing fluid [29]. Mao et al. prepared polyhydroxy gemini surfactant VES-m using UC22AMPM. 1% KCl was added into 5% VES-m surfactant solution as anti-ion salt to shield electrostatic repulsion, and the prepared VES clean fracturing fluid had a temperature resistance of 139 °C [31]. Zhang et al. designed sulfonate gemini surfactant EDBS composed of super-long hydrophobic chain, benzene ring and sulfonic acid by rigid–flexible combination strategy. The VES clean fracturing fluid showed good temperature resistance and shear resistance at 170 s^−1^ and 120 °C [32]. Cun et al. synthesized a new surfactant SY-JS by introducing a rigid cyclohexane structure into the molecule. The clean fracturing fluid prepared by 3 wt% SY-JS and 130,000 ppm NaCl or KCl showed good performance at 170 s^−1^ and 140 °C. The apparent viscosity remained above 30 mPa·s after 120 min [33]. However, there are few reports on VES clean fracturing fluid with temperature resistance exceeding 160 °C. Developing ultra-high temperature-resistant viscoelastic surfactant gel has great significance for expanding the application of VES fracturing fluid in extreme reservoir conditions.

**Figure 2:**
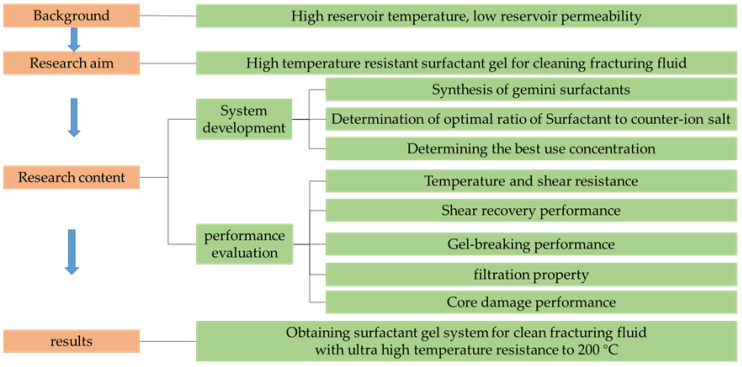
Research flow chart.

The research route of this paper is shown in Figure 2. In this work, a gemini surfactant 3-hydroxy-pentyl-distearamidopropyl dimethyl ammonium chloride named GOHAC containing hydroxyl and amide groups was synthesized by one-step method, and its structure was characterized. The ultra-high temperature-resistant surfactant gel was prepared with GOHAC as the thickener, and sodium *p*-toluenesulfonate (NaPts) was added into the GOHAC solution as the counter-ion salt, which was beneficial to the formation of surfactant gel. The effect of organic anti-ion salts on the formation of surfactant gel was investigated by studying the rheological properties of micelles formed by GOHAC and NaPts at different ratios. Under the optimum ratio of GOHAC/NaPts, the temperature resistance and shear recovery properties of ultra-high temperature viscoelastic surfactant gel were studied. It is expected to provide important information for the research and development of ultra-high temperature-resistant VES clean fracturing fluid.

## 2. Results and Discussion

### 2.1. Structure Characterization

**Figure 3:**
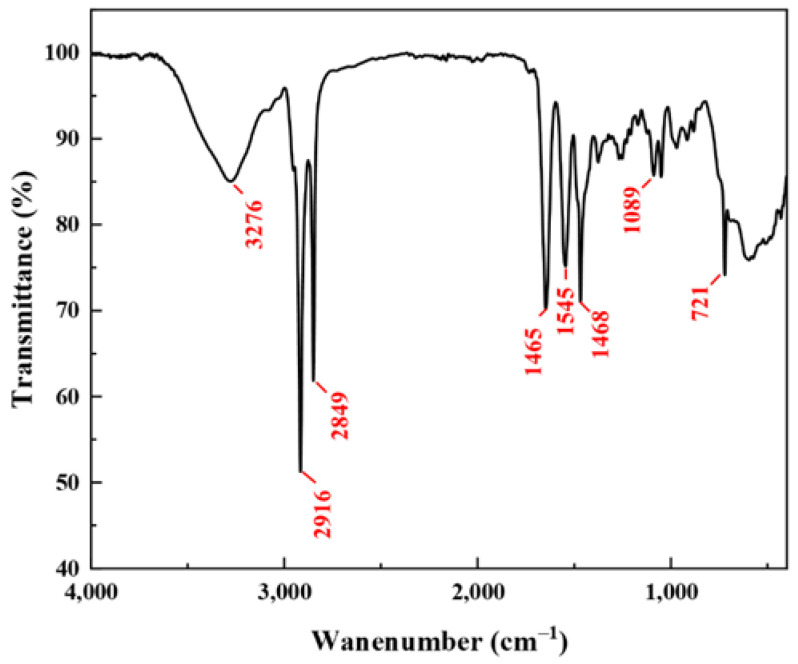
FTIR spectrum of GOHAC.

Fourier-transform infrared spectroscopy (FTIR) is one of the important methods to characterize the molecular structure of organic compounds [33]. The FTIR of the prepared gemini cationic surfactant GOHAC is shown in Figure 3. Remarkably, the absorption peak resulting from O-H is presented at 3276 cm^−1^, and the absorption peaks near 2916 cm^−1^ and 2849 cm^−1^ correspond to the stretching vibration absorption peaks of C-H. The strong absorption peak near 1649 cm^−1^ corresponds to the stretching vibration absorption peak of C=O on the amide group. The absorption peak at 1545 cm^−1^ is due to the N-H bending vibration absorptions on the secondary amide group. The C-N stretching peak appears at 1468 cm^−1^. Moreover, the peak at 1089 cm^−1^ corresponds to C-O stretching vibration and the peak at 721 cm^−1^ is the plane swing vibration absorption peak of (CH_2_)_n_.

**Figure 4:**
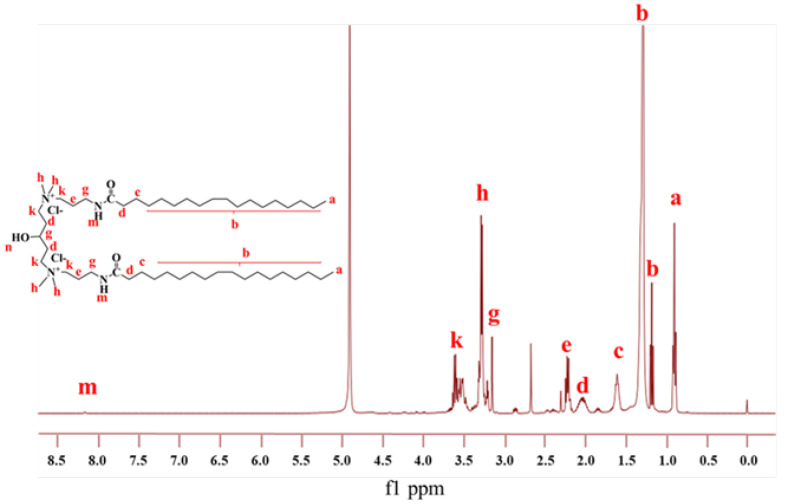
^1^H NMR spectrum of GOHAC.

^1^H NMR spectra are an important method to detect the structure of organic compounds, which is an important supplement to FTIR [34]. The 1H-NMR spectra of GOHAC surfactant are shown in Figure 4. The 1H-NMR (Methanol-d4) data are shown as follows: δ: 3.65–3.44 (m, 8H), 3.27 (d, J = 6.3 Hz, 12H), 3.23–3.08 (m, 3H), 2.26–2.14 (m, 4H), 1.60 (p, J = 7.3 Hz, 4H), 1.31 (q, J = 6.4, 4.3 Hz, 19H), 1.28 (s, 33H), 1.17 (t, J = 7.0 Hz, 3H) and 0.94–0.86 (m, 6H).

### 2.2. Surface Activity

**Figure 5:**
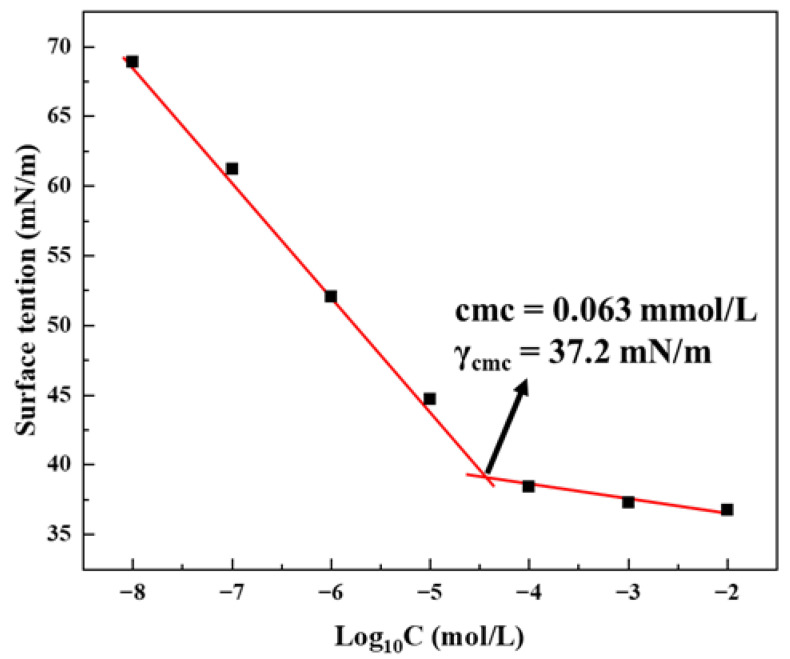
Surface tension plot for GOHAC.

The aggregation behavior of GOHAC in ultrapure water at 25 °C was investigated using the interfacial rheometer. Figure 5 shows the surface tension of GOHAC aqueous solution at different concentrations. The results showed that the surface tension decreased with the increase in surfactant concentration. When the surfactant concentration was relatively low, the surfactant could adsorb on the gas/liquid interface. With the increase in surfactant concentration, the surface tension was decreased continuously. When the surfactant concentration reached a certain value, the surface tension value gradually remained constant. This turning point corresponded to the critical micelle concentration (cmc) value of GOHAC. It can be seen from the figure that cmc value was 0.063 mmol/L, and the corresponding surface tension was 37.2 mN/m. When surfactant concentration is higher than cmc, spherical micelles begin to form in the solution [35].

### 2.3. Rheological Properties

#### 2.3.1. Steady Shear Properties

**Figure 6:**
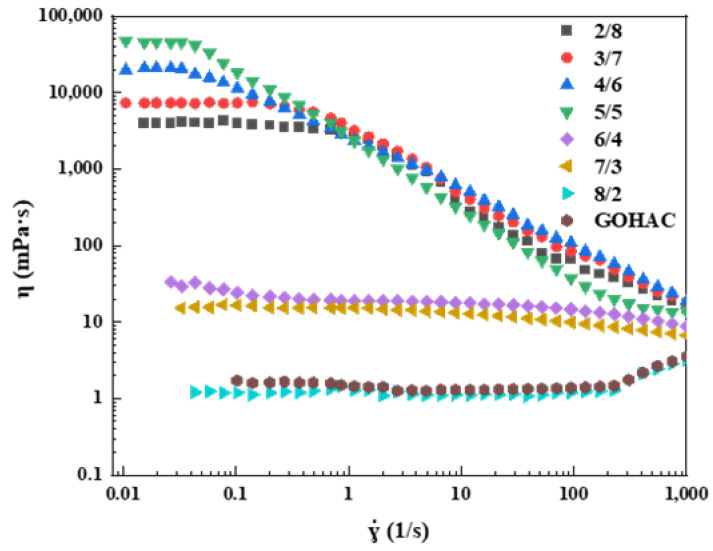
Steady shear viscosity of GOHAC/NaPts gel solution at different radios (the total concentration of GOHAC and NaPts is 40 mmol/L).

**Figure 7:**
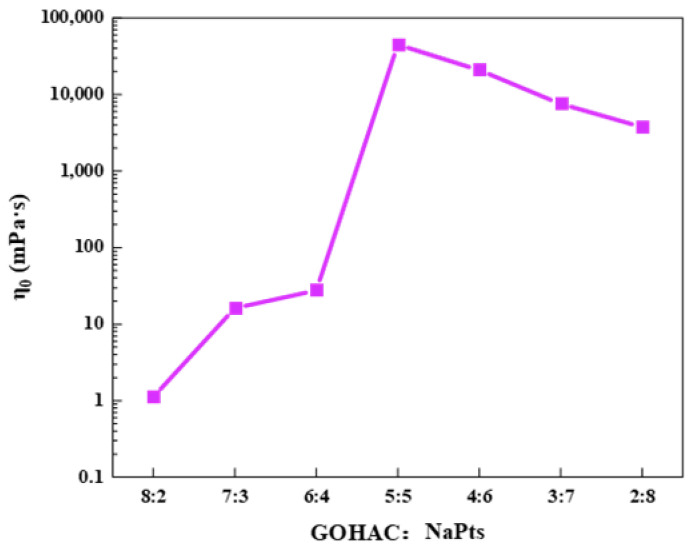
*η*_0_ of GOHAC/NaPts gel solution at different radios (the total concentration of GOHAC and NaPts is 40 mmol/L).

The steady shear properties of GOHAC and NaPts at different ratios were measured by fixing the total concentration of GOHAC and NaPts at 40 mmol/L, as shown in Figure 6. When the solution did not contain NaPts or contained less NaPts (GOHAC/NaPts = 8/2, 7/3, 6/4), the viscosity of the solution was close to that of water, and it was independent of the shear rate in the range of 0.01–1000 s^−1^. This was consistent with the characteristics of Newtonian fluid, indicating that there were only spherical micelles in the solution [36]. With the increase in NaPts content, the viscosity of the solution increases rapidly and the solution exhibits the characteristics of non-Newtonian fluid. Viscosity did not change with shear rate at low shear rate. This plateau value of viscosity can be regarded as zero-shear viscosity (*η*_0_) [37,38]. When the shear rate continued to increase, the system exhibited the typical characteristic of non-Newtonian fluid (shear thinning phenomenon). Figure 7 shows *η*_0_ at different ratios of GOHAC/NaPts. When the molar ratio of GOHAC/NaPts was 5:5, the *η*_0_ of the system reached 45 Pa·s. Compared with the pure GOHAC surfactant solution without NaPts, the *η*_0_ value increased by four orders of magnitude, indicating that the micelles grew rapidly along the one-dimensional direction [39,40,41]. The proportion of NaPts in the system continued to increase, and it can be seen that the *η*_0_ begins to decline. The main reasons include two aspects: (1) micellar branching: intermicellar junctions formed by micelle branches can glide along the worm and act as the stress release point and the entanglement point between micelles and the elasticity of solution were reduced [38]. (2) Space steric hindrance: excessive NaPts can also enhance the space steric hindrance between the benzene ring and micelles, which is not conducive to the formation of network structure [30].

#### 2.3.2. Dynamic Viscoelasticity

**Figure 8:**
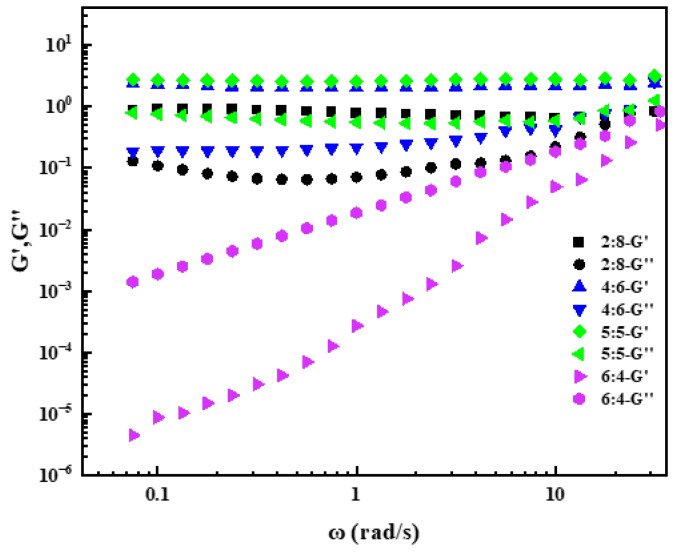
Dynamic viscoelasticity of GOHAC/NaPts gel solution at different radios (the total concentration of GOHAC and NaPts is 40 mmol/L).

Figure 8 showed the dynamic viscoelastic curves at different ratios of GOHAC/NaPts. It can be seen that the *G*′ and *G*″ were very low when the NaPts content was less than GOHAC. *G*″ was greater than *G*′ within the frequency range of the test. Moreover, the *G*′ and *G*″ increased with the increase in oscillation frequency in the range of 0.1–100 rad/s, indicating that the micelle structure at this time showed a relative dependence on frequency. In addition, it was not difficult to find that, with the increase in NaPts, *G*′ and *G*″ increased at first and then decreased, which was consistent with the change trend of *η*_0_ of the system. When GOHAC/NaPts = 5/5, the *G*′ and *G*″ of the system had the highest values. In addition, *G*′ is greater than *G*″ within the frequency range of the test, which indicated that the system exhibited gel properties. The above experimental results showed that, when GOHAC/NaPts = 1:1, the surfactant gel system formed at this time has the best viscoelasticity [42]. Therefore, the optimal ratio of GOHAC/NaPts was determined to be 1:1.

#### 2.3.3. High-Temperature Thermal Stability

**Figure 9:**
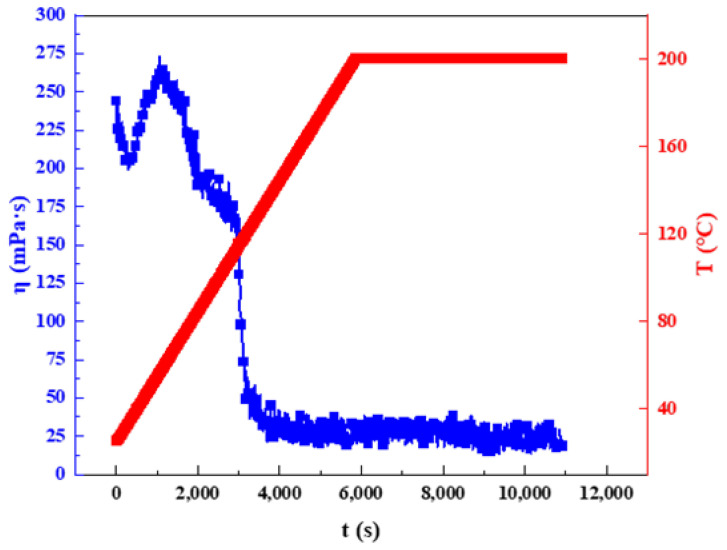
High-temperature thermal stability of GOHAC/NaPts gel solution (the total concentration of GOHAC and NaPts was 40 mmol/L, respectively).

The viscosity of clean fracturing fluid needs to reach a certain standard to meet the requirements of sand carrying in site construction [43]. Good sand-carrying performance can ensure that the fracture does not close after fracturing [44]. In general, during the flow of fracturing fluid from the ground to the target formation, the electrostatic interaction and hydrophobic association within the surfactant gels will continue to weaken due to the increase in formation temperature, resulting in a rapid decrease in the viscosity of surfactant gels [45]. Therefore, the high-temperature thermal stability of viscoelastic surfactant gel is particularly important. The high-temperature thermal stability of the gel solution was investigated at 170 s^−1^, as shown in Figure 9. As the temperature increased, the network structure in the system began to collapse, presented as a decrease in the viscosity of the system. The final test results showed that the viscosity of the system at 200 °C was 25.2 mPa·s. Moreover, the viscosity of the system remained almost unchanged after continuous shear at 200 °C for 1 h, indicating that the fracturing fluid had good high-temperature thermal stability and good sand-carrying capacity. The maximum applicable temperature of high-temperature-resistant clean fracturing fluid developed by relevant researchers is about 150–180 °C [12,25,46]. Compared with similar clean fracturing fluid, the maximum applicable temperature of the surfactant system prepared in this paper increased by more than 20 °C.

#### 2.3.4. Shear Recovery

**Figure 10:**
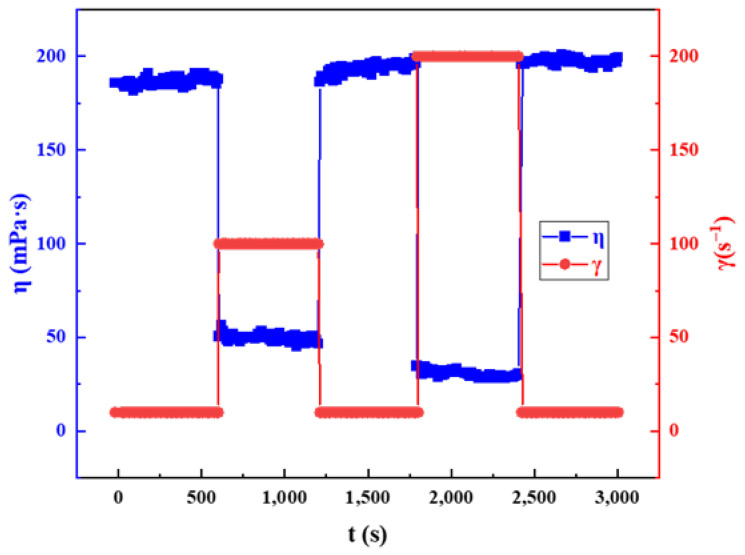
Shear recovery of GOHAC/NaPts gel solution.

Shear recovery is one of the important indexes to evaluate fracturing fluid field application performance [47]. The viscoelastic surfactant gel solution in this work belonged to pseudoplastic non-Newtonian fluid. The viscosity change in the fluid was inevitable during the injection process. The rapid change in shear viscosity and poor recovery of the system are likely to cause the decrease in sand-carrying capacity of the system, so the shear recovery has become an important guarantee to ensure the successful completion of fracturing operation. In order to evaluate the shear recovery of the fracturing fluid system, two different shear rate combinations were used, 10 s^−1^/100 s^−1^ and 10 s^−1^/200 s^−1^, respectively. The shear time was set for 10 min at each shear rate to ensure the stability of the fluid viscosity test results. Furthermore, no recovery waiting time was set between the two different shear rates. The experimental results are shown in Figure 10. Under two different experimental conditions, the viscoelastic surfactant gel showed good shear recovery ability. At the shear rates of 10 s^−1^, 100 s^−1^ and 200 s^−1^, the apparent viscosity of the clean fracturing fluid was 178.5 mPa·s, 50.3 mPa·s and 24.7 mPa·s, respectively, which was consistent with the viscosity test results at steady shear. In addition, the viscosity of the system did not change in the shear time of 10 min, indicating that the system had good shear resistance. The system also showed obvious rapid shear recovery ability. Under the combination of 10 s^−1^/100 s^−1^ and 10 s^−1^/200 s^−1^ shear rates, the apparent viscosity of the fluid can be quickly switched. Whether the shear rate decreased rapidly from high to low or increased rapidly from low, the recovery of the apparent viscosity of the fluid had no obvious time dependence.

Shear recovery depends on the self-healing properties of wormlike micelles in viscoelastic surfactant gel. Under the condition of high-speed shear, the morphology of the micelles will undergo a rapid transition from wormlike to rod-like to spherical. When shearing slows or stops, the wormlike micelles quickly recover and form stable structural strength.

### 2.4. Gel-Breaking Performance

**Figure 11:**
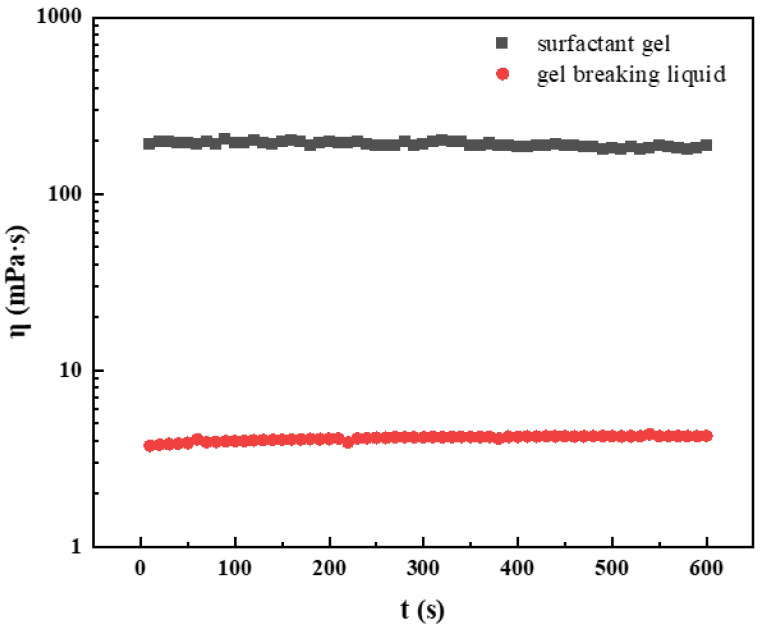
Viscosity variation of surfactant gel before and after breaking.

If the fracturing fluid exists in the form of gel in the reservoir after fracturing, it may block the fracture and matrix pores. Therefore, gel-breaking performance is one of the most important properties of fracturing fluid. For clean fracturing fluid, it is not necessary to add additional gel breaker, and gel breaking can be simply achieved in oil. Thus, for the gel breaking performance, 1 wt% kerosene as the gel breaker was mixed with surfactant gel to break the molecular structure of the fluid system. The test results are shown in Figure 11. It can be seen that 1% kerosene can completely break the molecular structure of the fluid system. The viscosity after fracturing was less than 5 mPa·s, meeting the industry standard of fracturing fluid performance.

### 2.5. Filtration Evaluation

**Figure 12:**
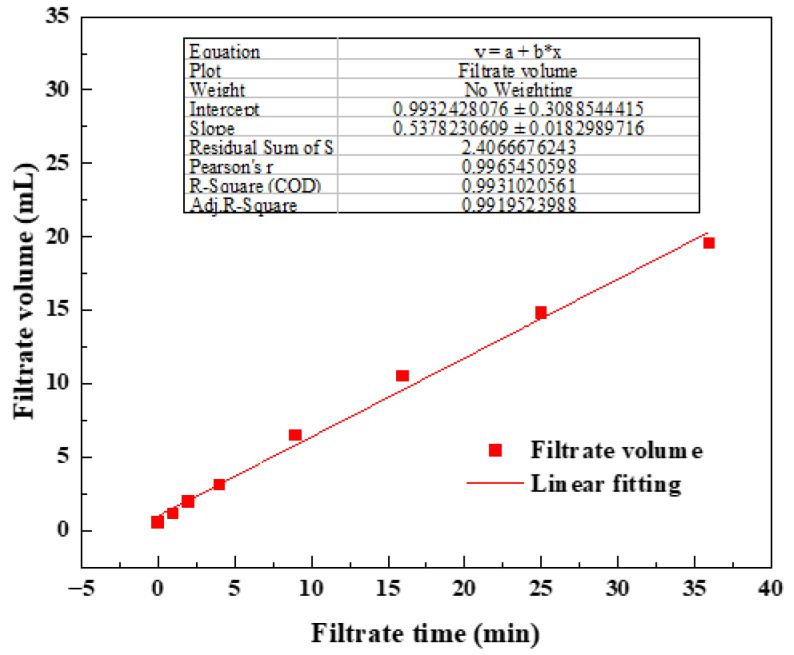
Filtration curves of surfactant gel.

Fluid loss can reduce the efficiency of fracturing, increase construction costs, and may even cause fracturing failure. Therefore, it is necessary to evaluate the filtration performance of the prepared surfactant gel. The experimental results are shown in Figure 12, which shows the filtration curve of the fluid at 10 mD core. According to the calculation, the filtration coefficient (*C*) was 2.9 × 10^−4^ m·s^−1/2^. The calculation results are shown in Table 1. The surfactant gel system had a small filtration loss, which met fracturing fluid performance industry standards.

### 2.6. Core Permeability Damage

In order to study the damage performance of surfactant gel as a clean fracturing fluid for reservoir permeability, we selected two low-permeability cores with similar initial permeability to conduct core permeability damage experiments of surfactant gel and conventional guanidine gum fracturing fluid, respectively. The results are shown in Table 2. It can be seen from Table 2 that the damage rate of fracturing fluid to low permeability cores was 6.23%. Compared with conventional guanidine gum fracturing fluid system, clean fracturing fluid system had less damage to core permeability. This indicated that the surfactant gel as a clean fracturing fluid had obvious advantages in reducing reservoir damage.

## 3. Conclusions

Recently, clean fracturing fluid has become very popular for recovery of low-permeability oil and gas fields. In this work, a novel surfactant gel for high-temperature-resistant clean fracturing fluid was developed with gemini cationic surfactant as the main agent in this work. The system was composed of GOHAC with the concentration of 40 mmol/L and the counter-ion NaPts with the concentration of 40 mmol/L.

Performance evaluation experiments showed that this surfactant gel had adequate rheology for respective fracturing applications under high temperature and high shear rate conditions. The maximum applicable temperature of prepared surfactant gel increased by more than 20 °C compared with similar clean fracturing fluid. The core permeability damage rate of the surfactant gel was reduced by about 80% compared with the commonly used guanidine gum fracturing fluid, proving that this new surfactant gel was a low-damage and formation-friendly fluid. The surfactant gel had the characteristic of self-breaking in oil. In addition, the filtration coefficient was 2.9 × 10^−4^ m·s^−1/2^, which met the industry standard of fracturing fluid performance evaluation.

We hope that the ultra-high temperature-resistant viscoelastic surfactant gel developed in this work can provide some support and guidance for the application of clean fracturing fluid in ultra-high temperature reservoir conditions.

## 4. Materials and Methods

### 4.1. Material

Ethanol (99.7%, Sinopharm Chemical Reagent Co., Ltd., Shanghai, China), N-(3-(dimethylamino)propyl)stearamide (OPA, 95%, Nantong Shajia Chemical Technology Co., Ltd., Nantong, China), 1,3-dichloro-2-propanol (98%, Shanghai Macklin Biochemical Co., Ltd., Shanghai, China), sodium p-toluenesulfonate (NaPts, 96%, Aladdin Co., Ltd., Shanghai, China) and ethyl acetate (99.5%, Aladdin Co., Ltd., Shanghai, China) were used as received.

### 4.2. Synthesis of GOHAC

**Figure 13:**
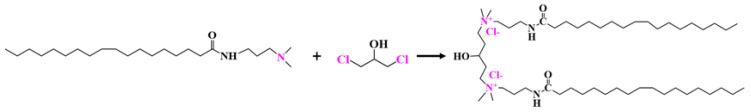
Synthesis principle of GOHAC.

GOHAC was synthesized by one-step quaternary ammonium method. The experimental principle is shown in Figure 13. The specific experimental steps were as follows:

Firstly, 151.24 g (0.205 mol) OPA and 75 mL ethanol were added into the 500 mL flask, and the flask was kept at 60 °C for a period of time to ensure the complete dissolution of OPA. Then, 25.8 g (0.2 mol) 1,3-dichloro-2-propanol was poured into the flask. The flask was heated by the oil bath at 90 °C with stirring for 24 h, and the stirring speed was set at 350 rpm. The flask was equipped with a condenser pipe. After the reaction, the solvent was removed by vacuum rotary evaporation. The product was washed with ethyl acetate to remove excess OPA. The final product GOHAC was obtained after vacuum drying for 24 h.

### 4.3. Structure Characterization

The final product GOHAC was characterized by ^1^H NMR using Bruker Advance 400M NMR spectrometers, and methanol-d4 was used as the solvent. In addition, the surfactant was analyzed by FTIR using Bruker Tensor-27 infrared spectrometer. The test samples were prepared by the potassium bromide (KBr) pellet method.

### 4.4. Viscoelastic Surfactant Gel Sample Preparation

Firstly, a certain amount of GOHAC was added into the ultrapure water. The solution was heated at 60 °C and stirred continuously to ensure the complete dissolution of GOHAC. Then, NaPts was added into GOHAC solution. With the addition of NaPts, the viscosity of the solution increases immediately. Finally, the prepared surfactant gel was stable for 24 h in an oven at 25 °C.

### 4.5. Surface Tension Measurement

The surface tension of the surfactant solution was measured by interfacial rheometer (Tracker, Teclis) and the experimental temperature was 25 °C. Before each measurement, ultrapure water was used to clean quartz dishes and syringes, and the surface tension of ultrapure water was tested at about 73 mN/m to ensure thorough cleaning. In this work, the surface tension of 10^−5^ mmol/L, 10^−4^ mmol/L, 10^−3^ mmol/L, 10^−2^ mmol/L, 10^−1^ mmol/L, 1 mmol/L and 10 mmol/L GOHAC solution was measured. The surface tension was measured three times and the average value was taken. The cmc value was obtained from the logarithmic relationship between surface tension and total surfactant concentration curve.

### 4.6. Rheological Measurement

The rheological properties of samples were investigated by Haake Mars 60 rheometer (Thermo Fisher, Karlsruhe, Germany) with coaxial cylinder rotor sensor system. Before measurement, samples were stable at a predetermined temperature for not less than 30 min. In the steady shear measurements, the range of shear rate was kept from 0.01 to 1000 s^−1^. In viscoelastic measurement, the scanning frequency was fixed firstly, and the stress scanning was carried out at a special frequency (*f* = 1 Hz) to determine the linear viscoelastic region. After that, the storage modulus (*G*′) and loss modulus (*G*″) of the system were measured at different oscillation frequencies. The oscillation frequency range was set to 0.01–100 rad/s.

### 4.7. High-Temperature Thermal Stability Evaluation Method

In this work, the high temperature stability of the samples was evaluated by the performance evaluation industry standard of water-based fracturing fluid (SY/T 5107-2016) [48]. The instrument used in the experiment was Haake Mars 60 rheometer (Thermo Fisher, Karlsruhe, Germany). The specific test method was as follows:

The shear rate was set to 170 s^−1^ and the heating rate was 1.8 °C/min. After reaching the target temperature, the viscosity change was observed after constant temperature shearing for 60 min. The industry standard requires that the viscosity of fracturing fluid should be at least 20 mPa·s to meet the requirements of fracturing fluid transportation proppants [25,44,49].

### 4.8. Gel-Breaking Performance Test

The 1 wt% kerosene was used to break the fracturing fluid. The shear viscosity was measured using a Haake MARS 60 (Thermo Fisher, Karlsruhe, Germany) Rheometer at 25 °C and a shear rate of 170 s^−1^ according to the fracturing fluid industry standards.

### 4.9. Filtration Test

**Figure 14:**
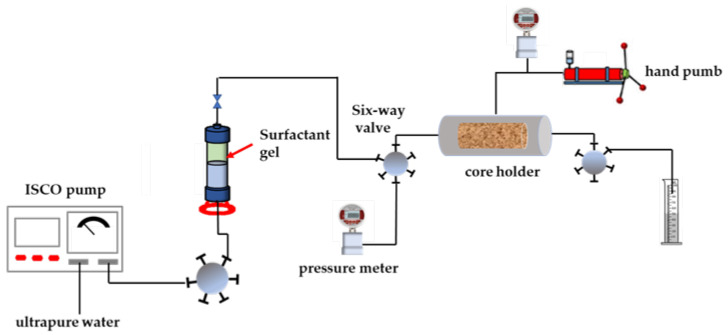
Filtration coefficient test system.

According to the industry standard of water-based fracturing fluid, the filtration coefficient of the surfactant gel was carried out. The experimental device is shown in Figure 14. Before the experiment, the porosity of the core was tested after the cleaning and drying procedure. Then, the core was placed into the core holder and the container filled with the surfactant gel was also placed into the oven to start the experiment. The filtration loss pressure was set at 6.895 MPa. The timing started when liquid began to flow out of the outlet end of the core holder. The filtrate volume was recorded at 1 min, 2 min, 4 min, 9 min, 16 min, 25 min and 36 min to facilitate subsequent calculation. Finally, the flow rate, filtration viscosity and filtration coefficient were calculated according to the following Formulas (1), (2) and (3), respectively:

(1) $$Q=\frac{V}{T\times 60}$$

where Q is the flow rate of the filtrate (cm^3^·s^−1^); V is the volume of the filtrate (cm^3^); and T is the time of the filtrate (min).

(2) $$\mu =\frac{kA\Delta P}{QL}\times 10$$

where μ is the viscosity of the filtrate (mPa·s); k is the permeability of the core (μm^2^); A is the cross-sectional area of the core (cm^2^); ΔP is the differential pressure between the core ends (MPa); and L is the length of the core (cm).

(3) $$C=\sqrt{\frac{k\varphi \Delta P}{2\mu }}$$

where C is the filtrate coefficient of fracturing fluid (m·s^−0.5^); φ is the porosity of the core (%); ΔP is the differential pressure between the core ends (Pa); and μ is the viscosity of the filtrate (Pa·s).

### 4.10. Core Permeability Damage Experiment

**Figure 15:**
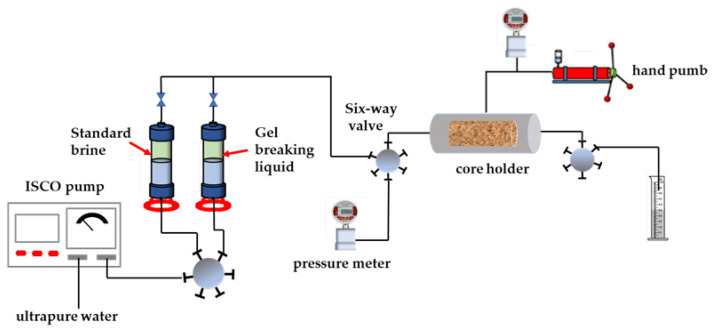
Core permeability damage test device.

According to the industry standard of water-based fracturing fluid, the core permeability damage experiment of the prepared fracturing fluid system was carried out. The experimental device is shown in Figure 15. The experimental steps were as follows:

(1) The core was treated to a preset size, cleaned with a KQ-300DE ultrasonic cleaner for 10 min, and dried in a 95 °C oven for 24 h. After fully drying, the core was taken out and cooled to room temperature.
(2) The standard brine with the concentration of 2.0 wt% KCl, 5.5 wt% NaCl, 0.45 wt% MgCl_2_ and 0.55 wt% CaCl_2_ was prepared. In addition, the core was put into the standard brine for vacuum saturation for 24 h.
(3) Preparation of gel breaking liquid: the surfactant gel is fully mixed with 1% kerosene and is stationary for 24 h, and the clear liquid is taken for use.
(4) Determination of initial permeability: a confining pressure of 2 MPa was applied around the core holder and the standard brine was displaced into the core by the ISCO pump at a flow rate of 0.5 mL/min. The stable pressure value was recorded, and the initial permeability was calculated according to formula (4).
(5) Simulating the damage process: the glue-breaking liquid was injected from the other end of the core at the same injection rate, with an injection volume of 1.5 PV. After the injection was completed, the valves at both ends of the core holder were closed so that the gel-breaking liquid stayed in the core for 2 h to simulate the soaking well process.
(6) Determination of permeability after damaging: the test steps were the same as Step 4.

Core permeability $k_{1},k_{2}$ were calculated according to the following formula:

(4) $$k=10^{-1}\times \frac{Q\mu L}{A\Delta P}$$

where k is the core permeability (mD), Q is the flow rate of the medium (cm^3^/s), *μ* is the viscosity of the medium (mPa·s), L is the core length (cm), A is the cross-sectional area of the core (cm^2^) and ΔP is the pressure difference at the inlet and outlet of the core (KPa).

The core damage rate was calculated according to the following formula:

(5) $$\eta_{D}=\frac{k_{1}-k_{2}}{k_{1}}\times 100\%$$

where $k_{1}$ is the damage rate (%), $k_{2}$ is the core permeability before damage (mD) and $\eta_{D}$ is the core permeability after damage (mD).

## Figures and Tables

**Table 1:** Calculation results of filtration coefficient.

*Q* (mL·min^−1^)	*A* (cm^2^)	*k* (m^2^)	*L* (cm)	*φ*	*C* (m·s^−1/2^)
0.54	4.91	1.21 × 10^−15^	4.97	19.35%	2.90 × 10^−4^

**Table 2:** Evaluation of core damage performance of the fracturing fluid system.

Core No.	Fracturing Fluid Type	Length (cm)	Diameter (cm)	Initial Permeability (mD)	Damage Permeability (mD)	Damage Rate (%)
1	Surfactant gel	5.01	2.50	3.85	3.61	6.23%
2	guanidine gum	4.98	2.50	3.79	2.63	30.61%

## Data Availability

Not applicable.
